# Supplementary material for: Genome-Wide Selection Scans in Mexican Indigenous Populations Reveal Recent Signatures of Pathogen and Diet Adaptation
Source: Genome Biol Evol. 2025 Mar 12;17(3):evaf043. doi: 10.1093/gbe/evaf043 (PMC11954594; doi:10.1093/gbe/evaf043)
Supplement: evaf043_Supplementary_Data [file evaf043_supplementary_data.zip › Supplementary_Figures.docx]

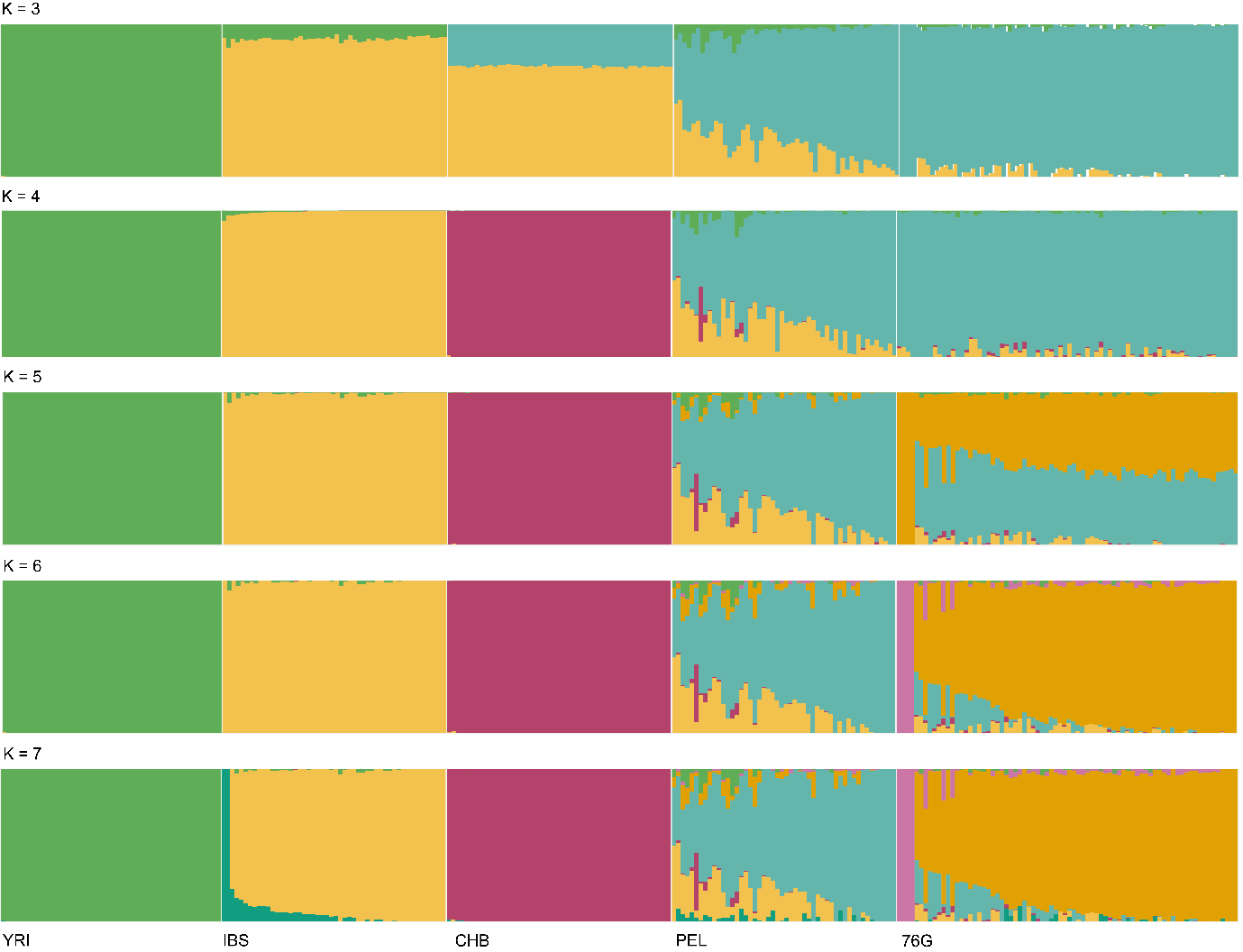


**Figure S1.** ADMIXTURE analysis for K=3-7 showing a high proportion of Native American ancestry on the 76 MI samples. References include Yoruba (YRI), Iberians (IBS), Han Chinese (CHB), and Peruvians (PEL) from the 1KGP.


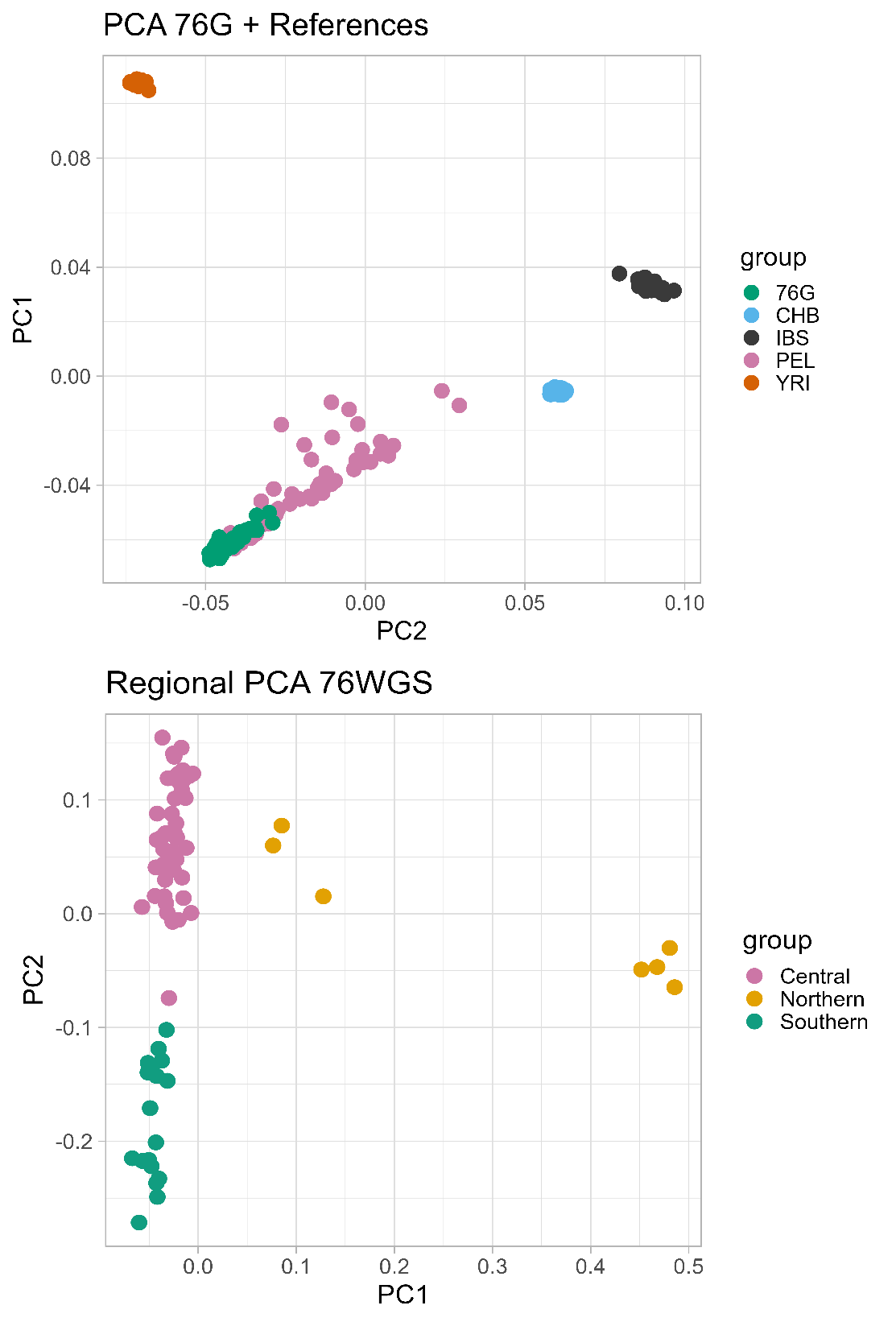


**Figure S2.** Principal Component Analysis exhibiting **A)** Global clustering of the 76G. References include Yoruba (YRI), Iberians (IBS), Han Chinese (CHB), and Peruvians (PEL) from the 1KGP. **B)** Local clustering of Northern, Central, and Southern Regions of the 76G.


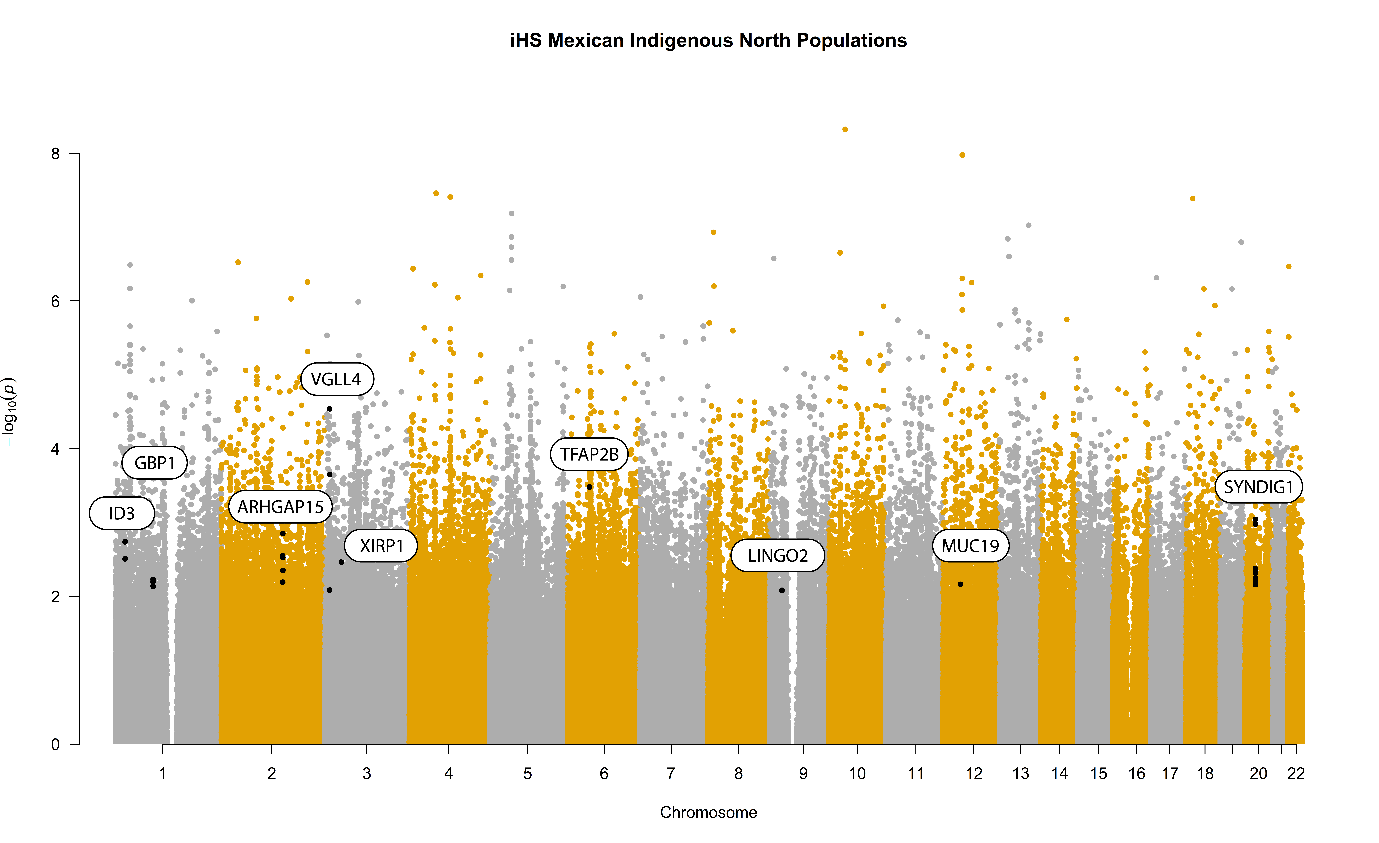


**Figure S3.** Manhattan plot of the iHS p-values for the Northern Mexican Indigenous Populations. Labels and black dots represent putatively selected genes discussed for the North MI region.


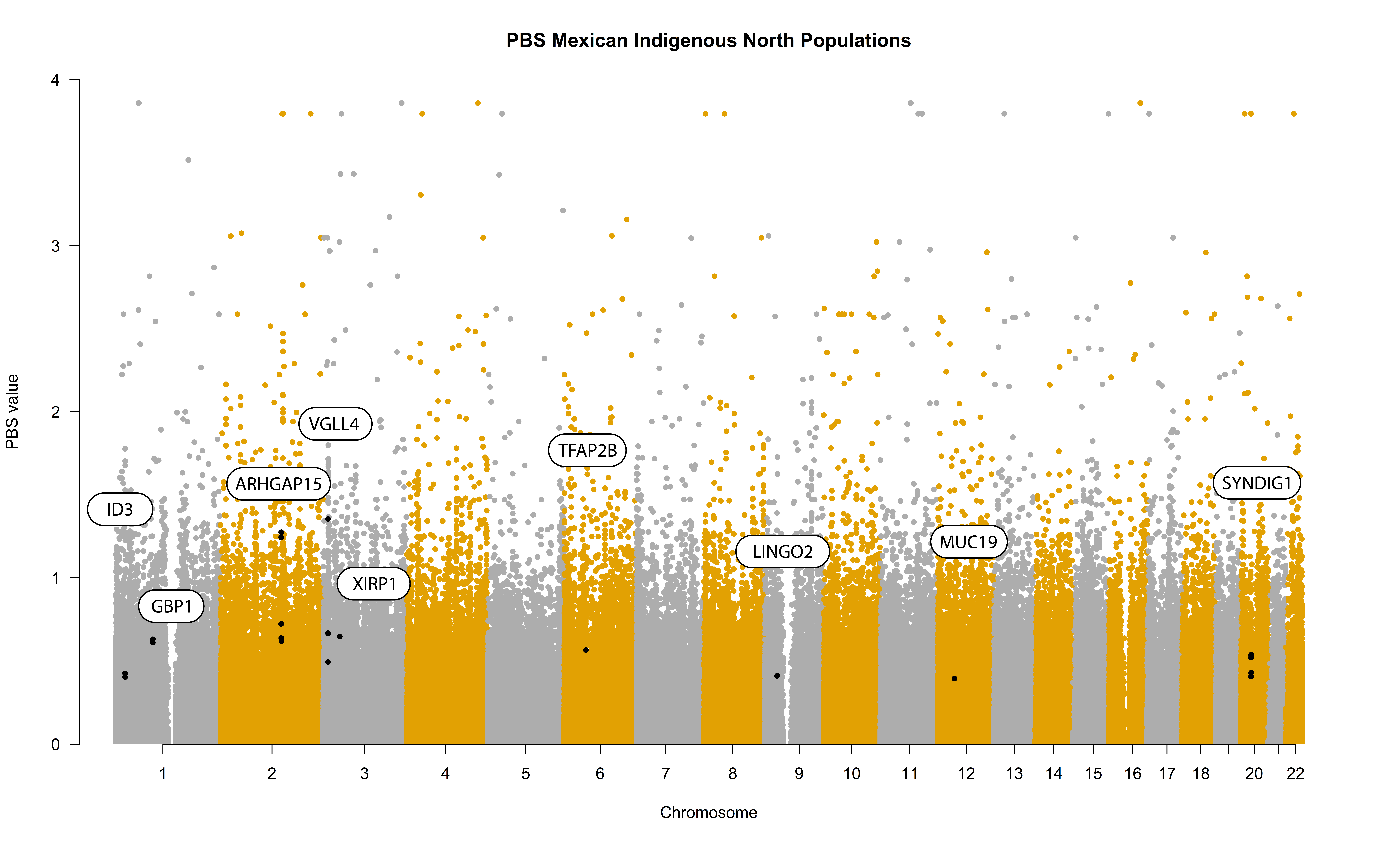


**Figure S4.** Manhattan plot of the PBS values for the Northern Mexican Indigenous Populations. Labels and black dots represent putatively selected genes discussed for the North MI region.


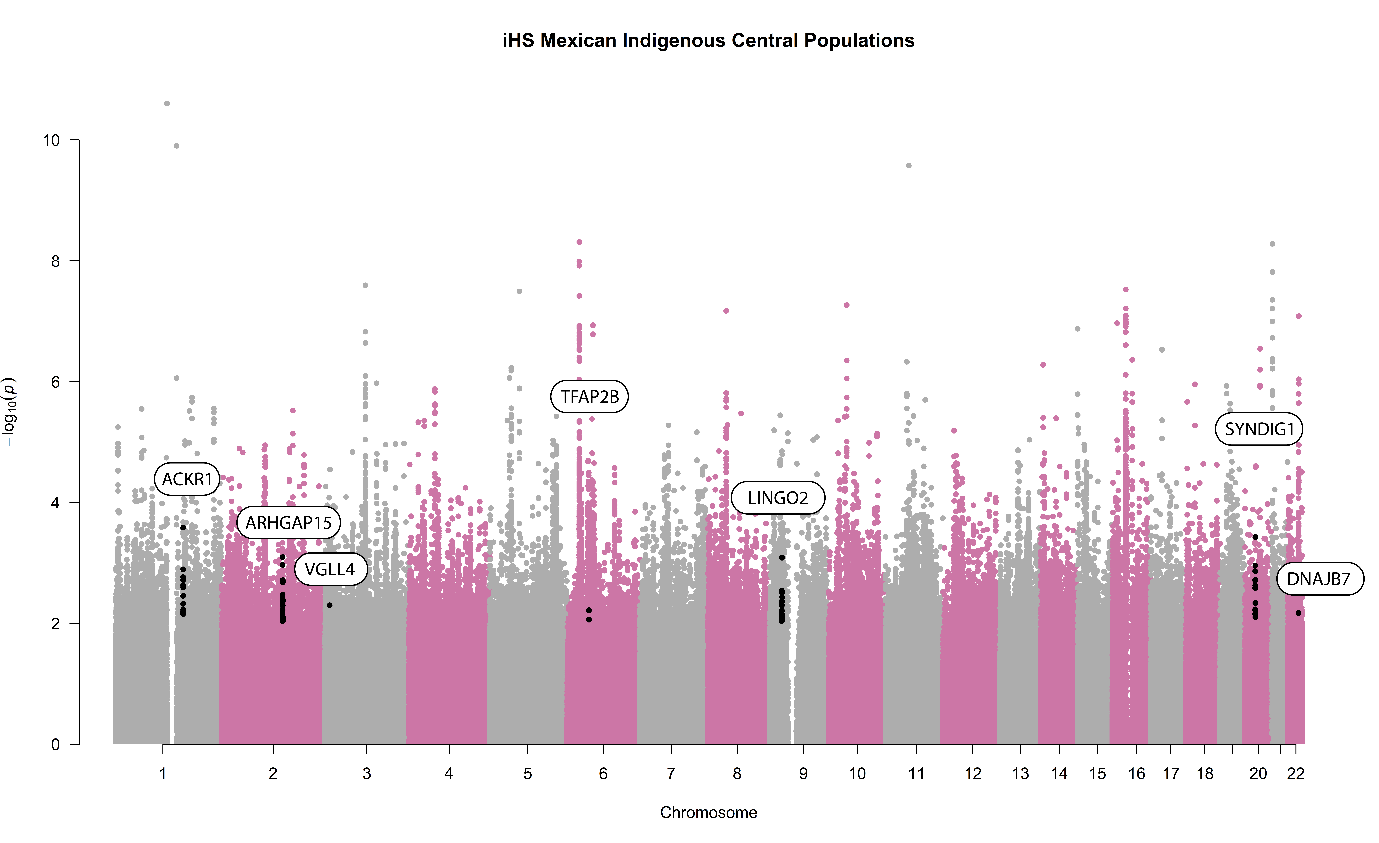


**Figure S5.** Manhattan plot of the iHS p-values for the Central Mexican Indigenous Populations. Labels and black dots represent putatively selected genes discussed for the Central MI region.


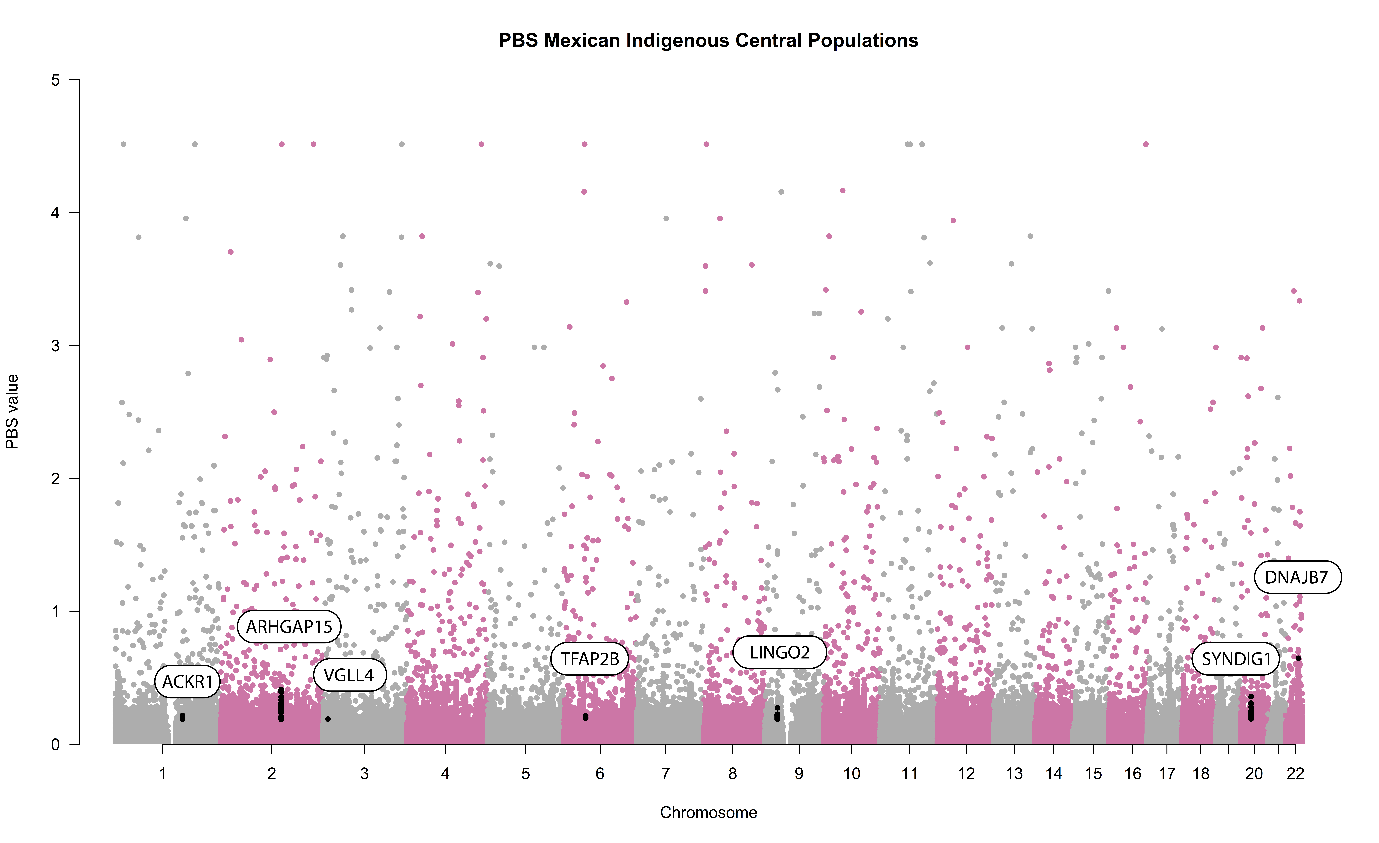


**Figure S6.** Manhattan plot of the PBS values for the Central Mexican Indigenous Populations. Labels and black dots represent putatively selected genes discussed for the Central MI region.


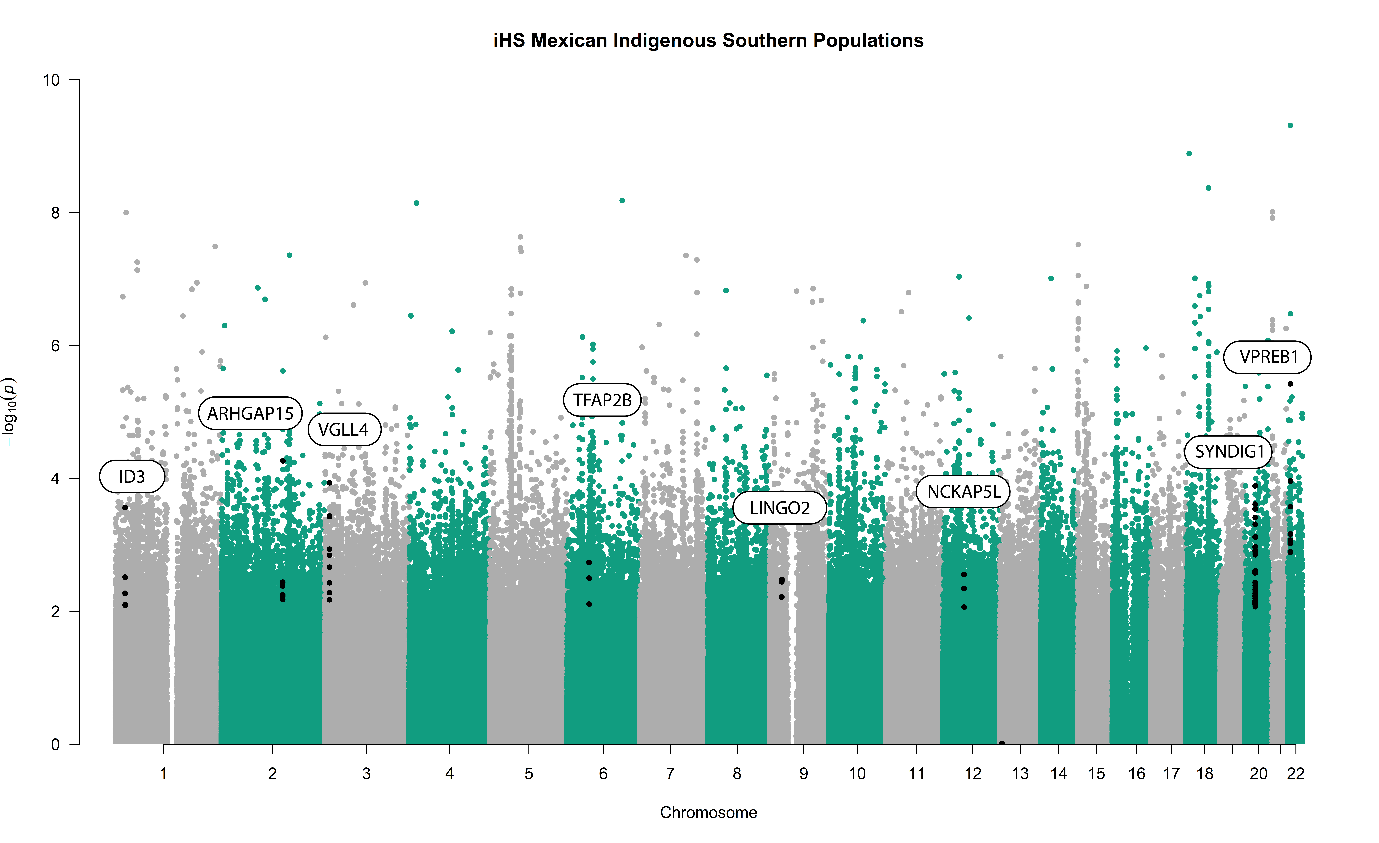


**Figure S7.** Manhattan plot of the iHS p-values for the Southern Mexican Indigenous Populations. Labels and black dots represent putatively selected genes discussed for the Southern MI region.


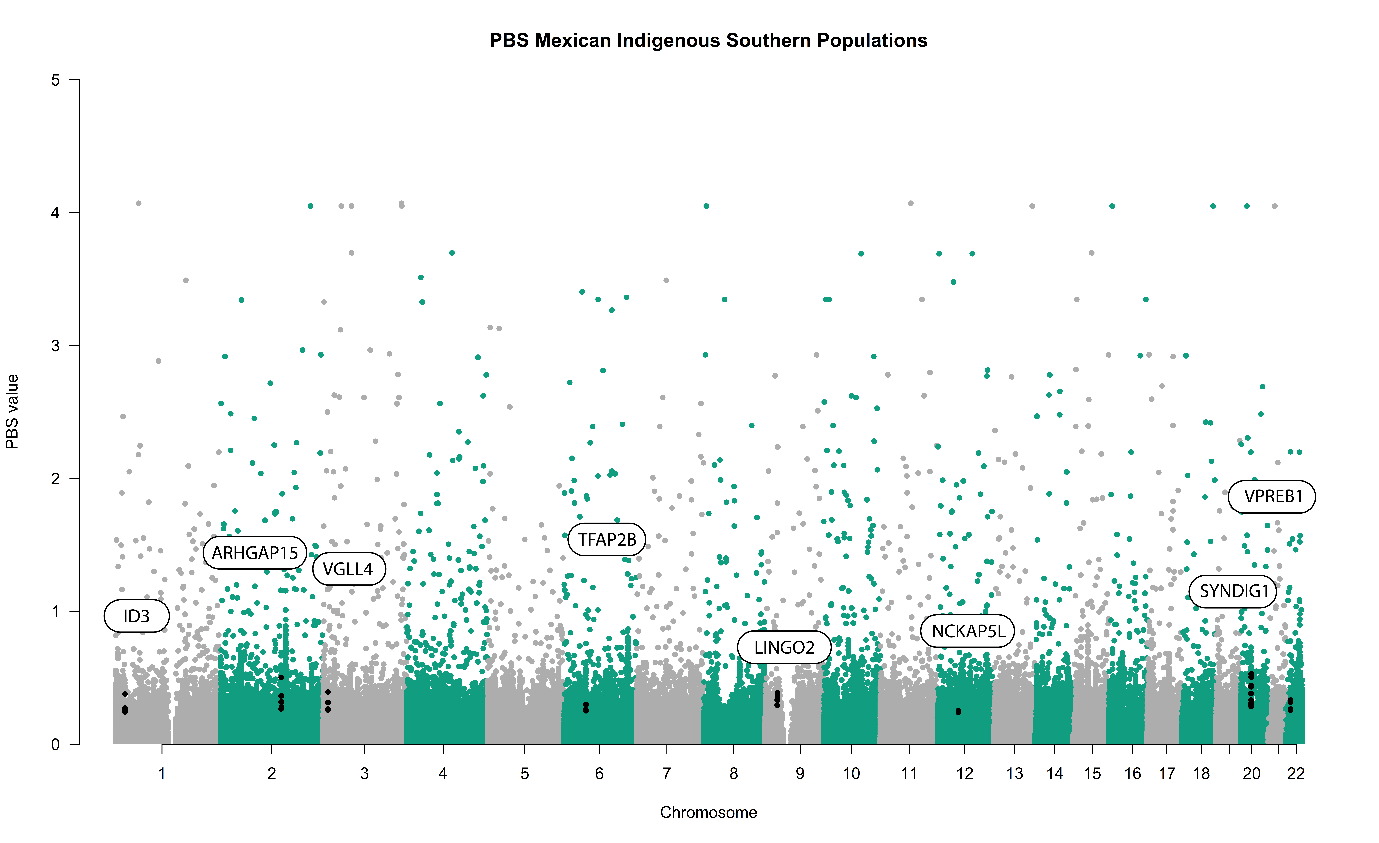


**Figure S8.** Manhattan plot of the PBS values for the Southern Mexican Indigenous Populations. Labels and black dots represent putatively selected genes discussed for the Southern MI region.


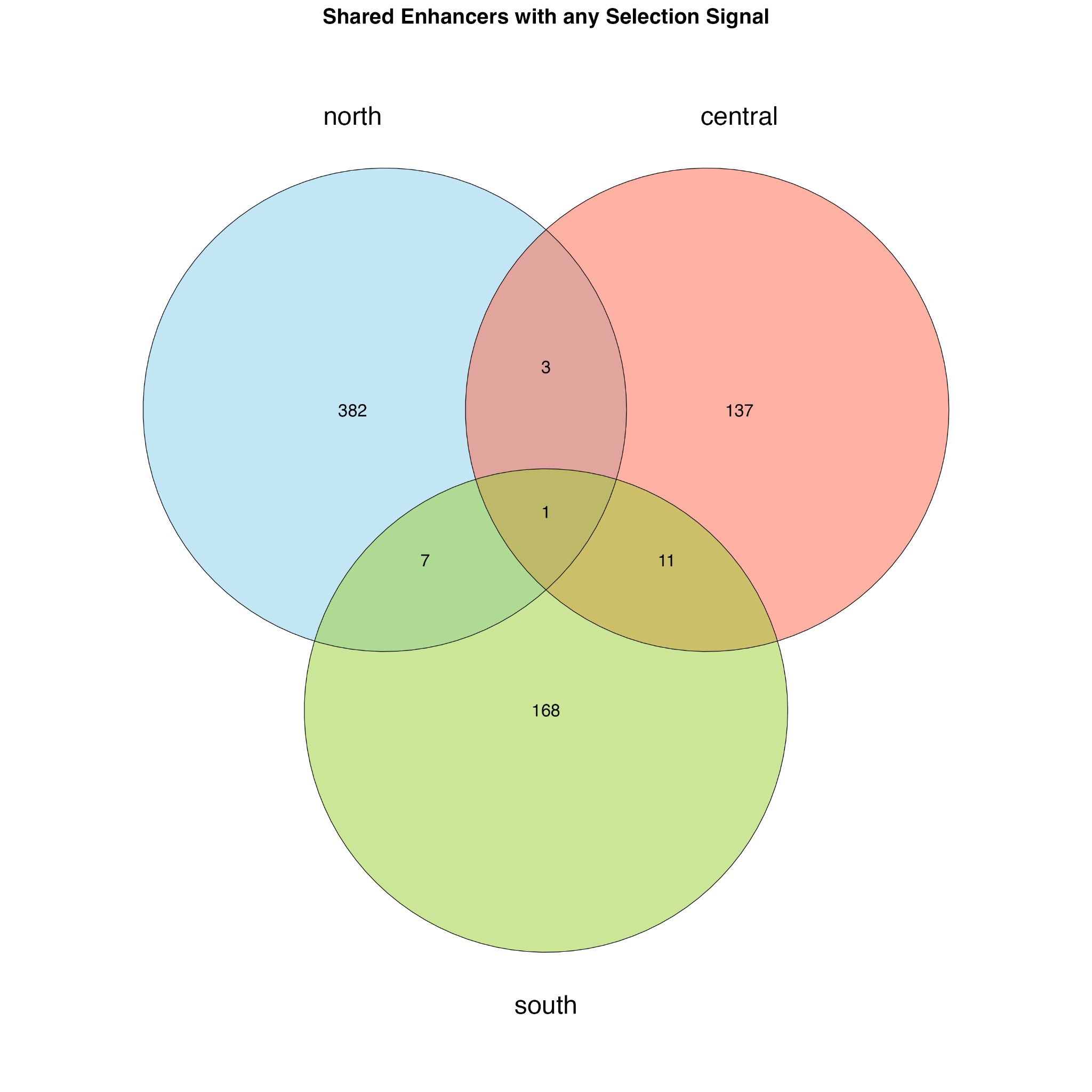


**Figure S9. Shared regulatory elements with selection signals.** Some enhancers are altered in more than one of the geographic regions, while some are exclusive to a region.

**
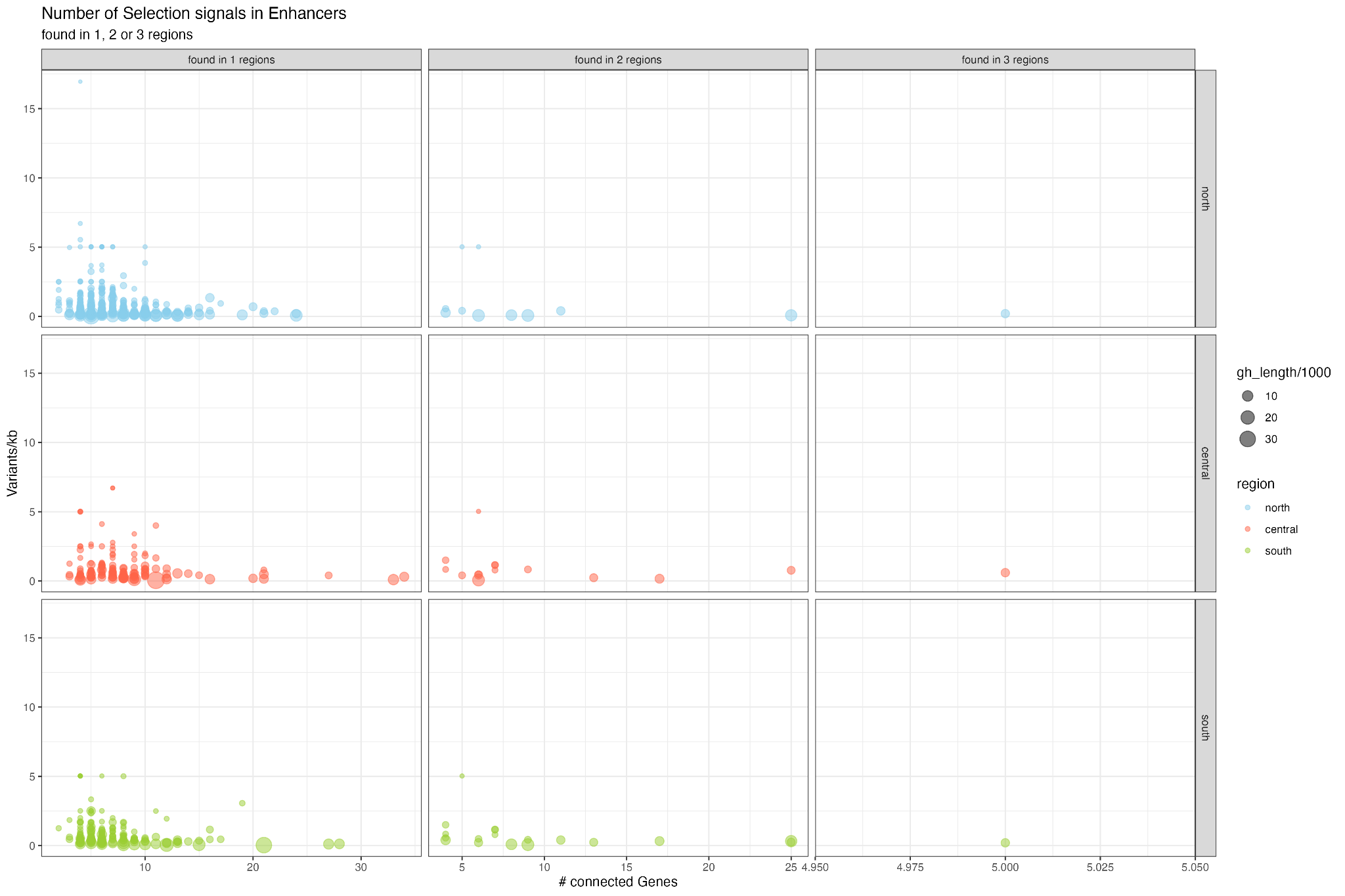
**

**Figure S10. Selection signal density by region.** We measured the number of selection signal SNPs divided by the length of the regulatory element (y-axis). We found that selection signals shared by 2 or 3 geographic regions show lower selection signal density.

**
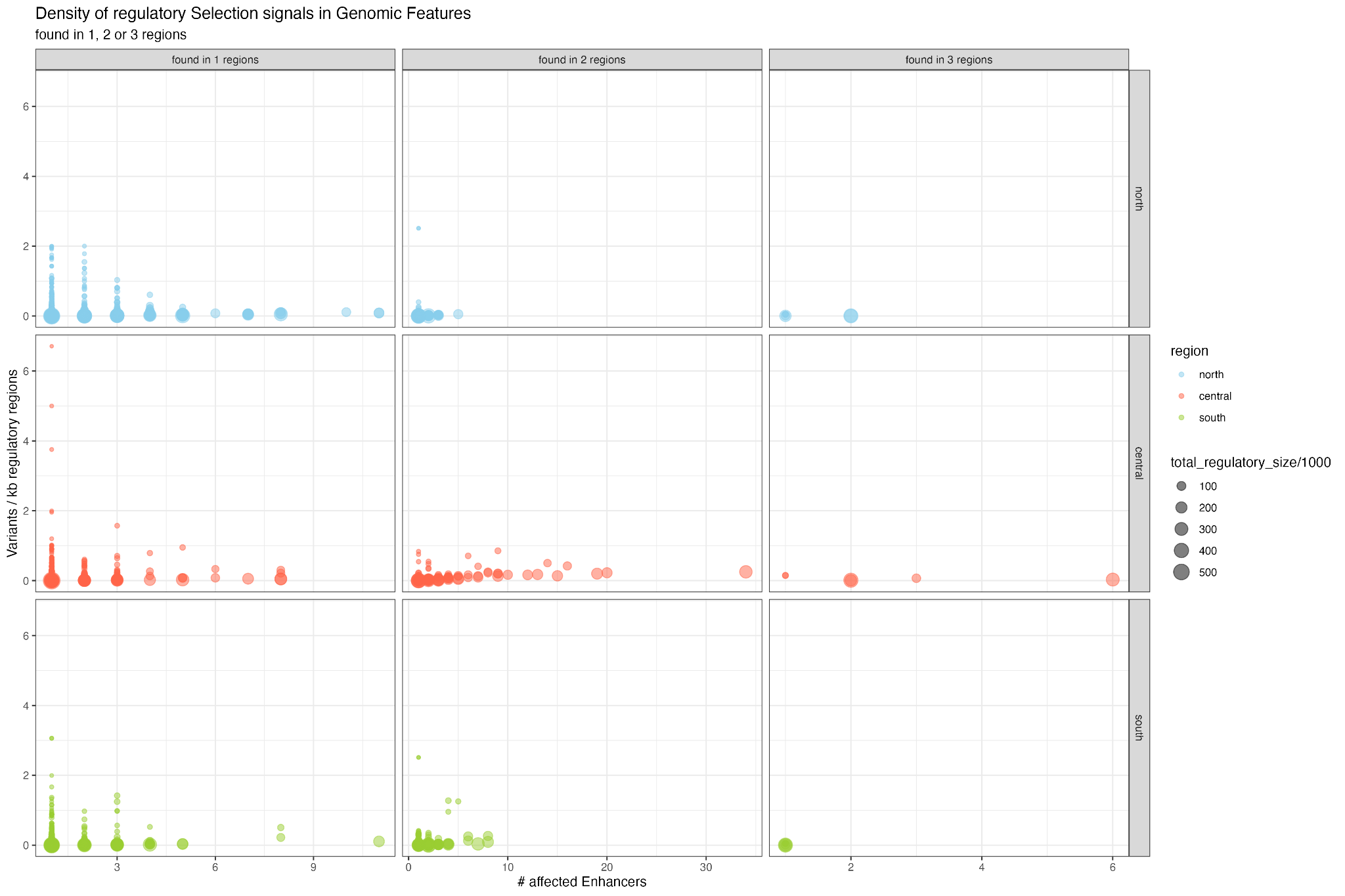
**

**Figure S11**. Regulatory selection signal density in genomic features by region. We measured the number of selection signal SNPs divided by the summarized length of all the regulatory elements for a given feature (y axis) and found that altered regulatory elements shared by 2 or 3 geographic regions show lower selection signal density.

**
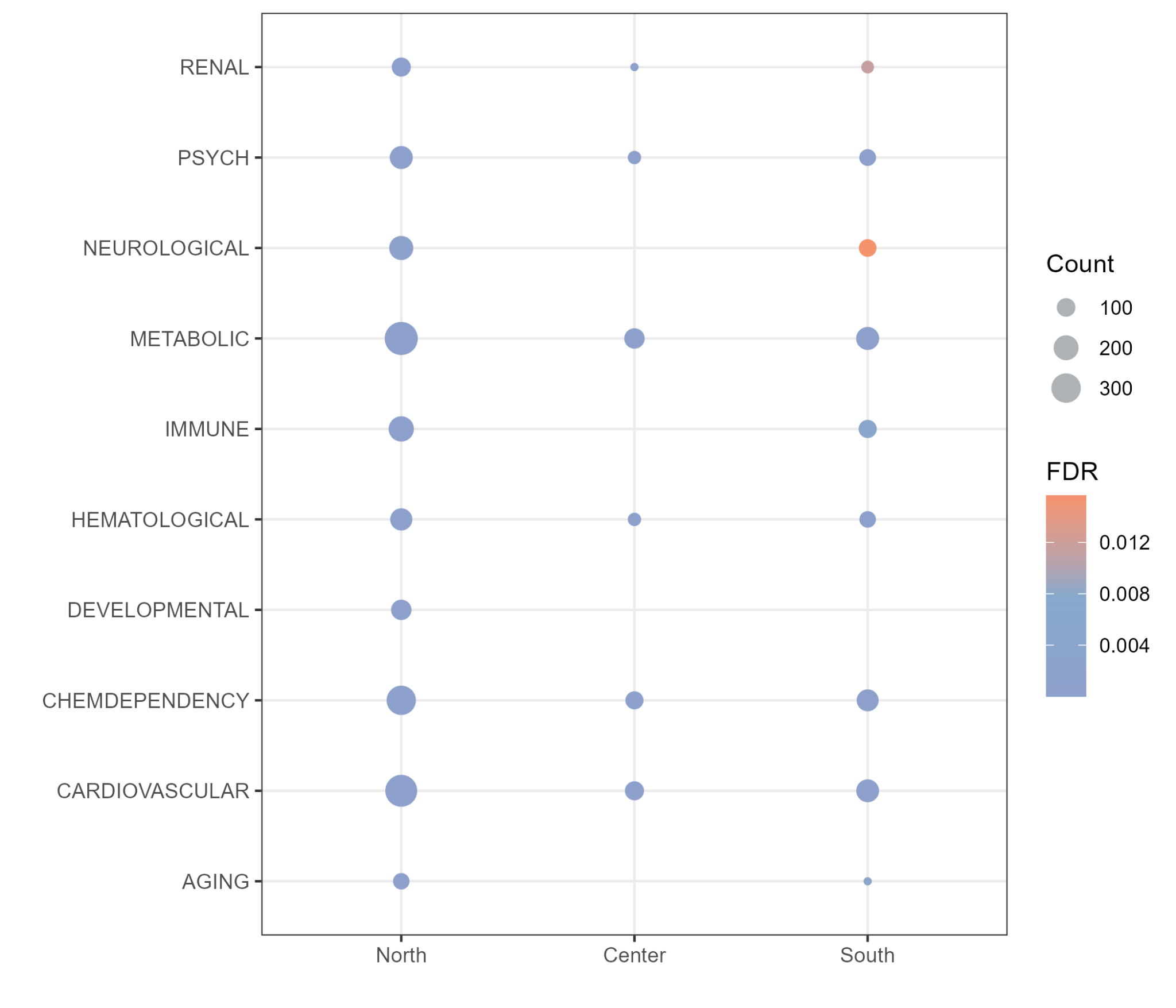
**

**Figure S12**. Gene disease association for Northern, Center, and Southern Mexican Indigenous Populations with DAVID. Terms represented in the analysis and gene count for each are depicted.


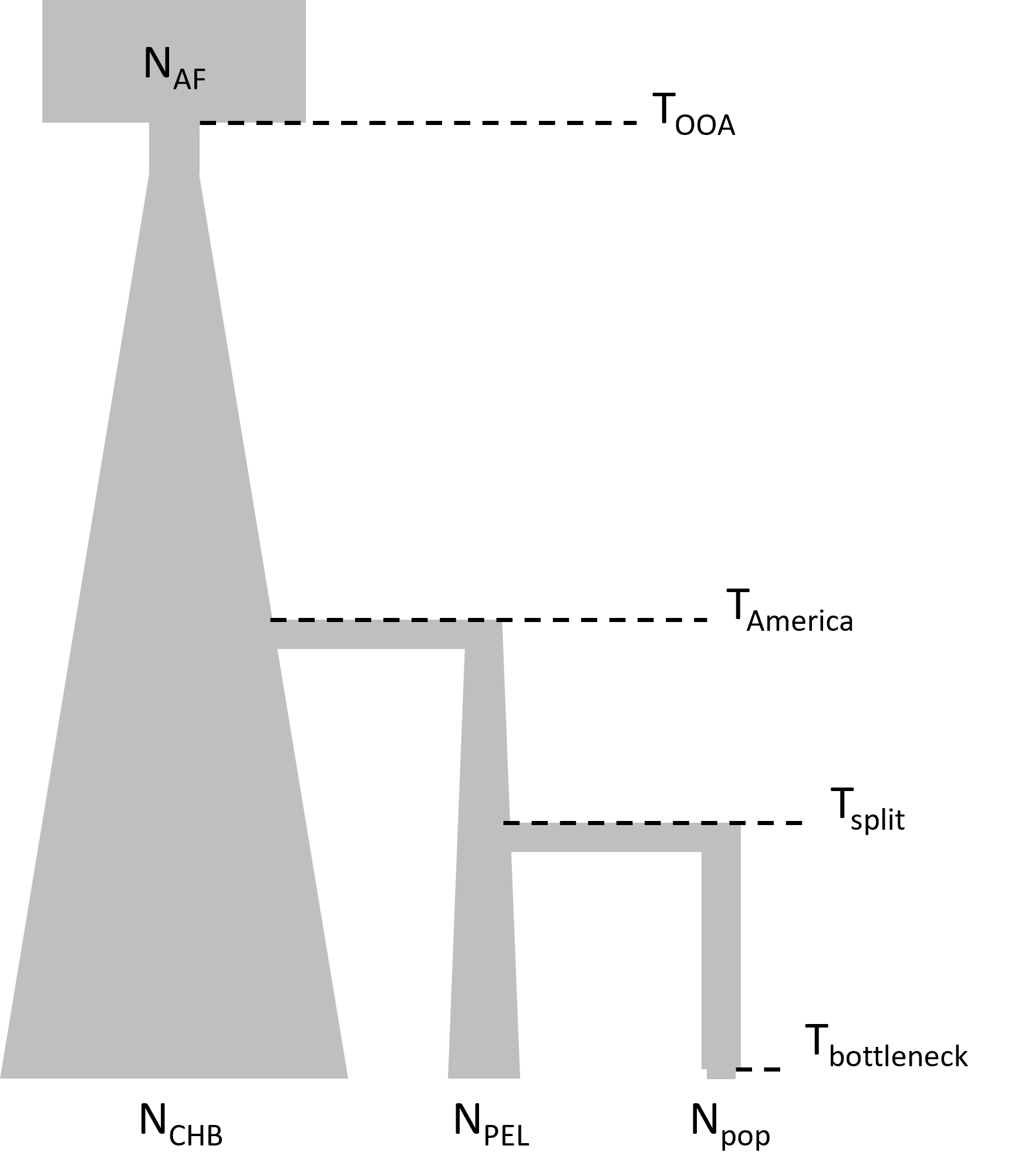


**Figure S13.** Demographic model for Mexican Indigenous Populations. Five demographic events were included. Time is given in generations.
